# Complement Genetic Variants and FH Desialylation in *S. pneumoniae*-Haemolytic Uraemic Syndrome

**DOI:** 10.3389/fimmu.2021.641656

**Published:** 2021-03-11

**Authors:** Irene Gómez Delgado, Fernando Corvillo, Pilar Nozal, Emilia Arjona, Álvaro Madrid, Marta Melgosa, Juan Bravo, Ágnes Szilágyi, Dorottya Csuka, Nóra Veszeli, Zoltán Prohászka, Pilar Sánchez-Corral

**Affiliations:** ^1^Complement Research Group, Hospital La Paz Institute for Health Research (IdiPAZ), La Paz University Hospital, Madrid, Spain; ^2^Center for Biomedical Network Research on Rare Diseases (CIBERER), Madrid, Spain; ^3^Immunology Unit, Hospital La Paz Institute for Health Research (IdiPAZ), La Paz University Hospital, Madrid, Spain; ^4^Department of Cellular and Molecular Medicine, Margarita Salas Center for Biological Research, Madrid, Spain; ^5^Pediatric Nephrology, Hospital Sant Joan de Déu, Barcelona, Spain; ^6^Pediatric Nephrology Unit, Hospital La Paz Institute for Health Research (IdiPAZ), La Paz University Hospital, Madrid, Spain; ^7^Research Laboratory, Department of Internal Medicine and Hematology, Semmelweis University, Budapest, Hungary; ^8^Research Group for Immunology and Haematology, Semmelweis University- Eötvös Loránd Research Network (Office for Supported Research Groups), Budapest, Hungary

**Keywords:** factor H, *Streptococcus pneumoniae* (pneumococcus), Haemolytic Uraemic Syndrome, genetic variant, complement system

## Abstract

Haemolytic Uraemic Syndrome associated with *Streptococcus pneumoniae* infections (SP-HUS) is a clinically well-known entity that generally affects infants, and could have a worse prognosis than HUS associated to *E. coli* infections. It has been assumed that complement genetic variants associated with primary atypical HUS cases (aHUS) do not contribute to SP-HUS, which is solely attributed to the action of the pneumococcal neuraminidase on the host cellular surfaces. We previously identified complement pathogenic variants and risk polymorphisms in a few Hungarian SP-HUS patients, and have now extended these studies to a cohort of 13 Spanish SP-HUS patients. Five patients presented rare complement variants of unknown significance, but the frequency of the risk haplotypes in the *CFH-CFHR3-CFHR1* region was similar to the observed in aHUS. Moreover, we observed desialylation of Factor H (FH) and the FH-Related proteins in plasma samples from 2 Spanish and 4 Hungarian SP-HUS patients. To analyze the functional relevance of this finding, we compared the ability of native and “*in vitro*” desialylated FH in: (a) binding to C3b-coated microtiter plates; (b) proteolysis of fluid-phase and surface-bound C3b by Factor I; (c) dissociation of surface bound-C3bBb convertase; (d) haemolytic assays on sheep erythrocytes. We found that desialylated FH had reduced capacity to control complement activation on sheep erythrocytes, suggesting a role for FH sialic acids on binding to cellular surfaces. We conclude that aHUS-risk variants in the *CFH-CFHR3-CFHR1* region could also contribute to disease-predisposition to SP-HUS, and that transient desialylation of complement FH by the pneumococcal neuraminidase may have a role in disease pathogenesis.

## Introduction

*Streptococcus pneumoniae* (SP) infections can give rise to potentially life-threatening infections such as pneumonia, meningitis or sepsis, especially in children under 2 years of age (1, 2). In the last 20 years, the generalization of vaccination against several SP serotypes have dramatically reduced the incidence and morbidity/mortality of these conditions, but many serotypes are not covered by vaccination and some are antibiotic-resistant (3).

Invasive *S. pneumoniae* infections sometimes result in a form of Haemolytic Uraemic Syndrome (SP-HUS) with high morbidity/mortality (1, 4, 5). It is thought that SP-HUS results from desialylation of host cells by the pneumococcal neuraminidase, which result in the exposition of the *Thomsen-Friedenreich* antigen (TF) in erythrocytes, platelets and glomeruli and its subsequent interaction with natural anti-TF antibodies (6, 7), and/or in reduced protection of host cells against autologous complement (8, 9). On the assumption that this is the main pathogenic mechanism, it is generally accepted that complement genetic variants are not involved in predisposition to SP-HUS (10). Nonetheless, we have already described a few SP-HUS patients presenting rare genetic variants in complement genes (11, 12), and these findings suggested that the complement contribution to SP-HUS could be underestimated.

Human Complement can eliminate *S. pneumoniae* through different mechanisms (13). *S. pneumoniae* activates the complement classical pathway, as illustrated by the high incidence of infections in individuals with deficiency of C1q, C2, or C4 (14). Nonetheless, the lectin and alternative pathways also contribute to bacterial killing, which is mainly done through opsonophagocytosis, and to a lesser extent through inflammation. Assembly of the Membrane Attack Complex on the pathogen surface, on the contrary, is of little relevance because of the presence of the *S. pneumoniae* capsule, which is a very important virulence factor and the first barrier against the immune system proteins.

*S. pneumoniae* can also avoid elimination by human complement by expressing several proteins (PspC, SpsA, Hic, C3-binding protein) that interact with human Factor H (FH), the main regulator of the complement alternative pathway (15, 16). FH is a 150-kDa plasma glycoprotein that is essential to control complement activation on plasma and on cellular surfaces, thus preventing hypocomplementemia and self-damage (17). The complement regulatory activities of FH rely on its interaction with soluble or surface-bound C3b, and with negatively-charged molecules (mainly sialic acids and glycosaminoglycans) present on host's cells and tissues. The distribution of ligand-binding sites in FH is well-known. The N-terminal, SCRs 1-4 domains bind to soluble C3b, SCRs 6-7 recognize soluble or surface polyanions, and the C-terminal, SCRs19-20 domains recognize both C3b and polyanions on cellular surfaces (18, 19). Defective function of the N-terminal domains of FH provokes uncontrolled complement activation in plasma and deposition of C3b fragments on autologous cells and tissues, thus favoring renal pathologies such as membranoproliferative glomerulonephritis. Defective function of the C-terminal domains of FH, in the other hand, predominantly alter complement regulation on cellular surfaces, and contributes to the endothelial damage characteristic of the thrombotic microangiopathy atypical HUS (aHUS) (20). FH function could be modulated by their homologous FH-Related (FHR) proteins, a group of plasma proteins whose precise role on complement physiopathology is not fully understood (21, 22).

In this report, we extend our complement findings to a cohort of 13 Spanish SP-HUS patients. We confirm the presence of rare complement genetic variants in SP-HUS patients, and show that there is a high frequency of some FH and FHRs polymorphisms associated to aHUS. Moreover, we report for the first time the transient desialylation of FH and FHR proteins by the pneumococcal neuraminidase in plasma samples from a few Spanish and Hungarian SP-HUS patients, and present functional data suggesting that FH sialic acids have a certain role in complement regulation on cellular surfaces.

## Materials and Methods

### Blood Samples

Blood samples from 13 Spanish and 11 Hungarian SP-HUS patients were drawn during the acute episode or at remission. EDTA-plasma was aliquoted and stored at −20 and −80°C until use, to avoid repeated freezing and thawing; peripheral blood leukocytes (PBLs) were used to prepare genomic DNA by standard procedures. Blood samples were also obtained from healthy volunteers. Patients and controls provided written informed consent, as approved by the ethical committees from La Paz University Hospital or the Semmelweis University.

### Genetic Studies

Mutational screening on the Spanish patients was determined by an in-house next generation sequencing (NGS) panel which includes all the complement genes relevant to aHUS (23). Copy number variation in the *CFH-CFHRs* region was analyzed by multiplex ligation-dependent probe amplification (MLPA) with the P236 A1 ARMD mix 1 (MRC-Holland, Amsterdam, The Netherlands). Genotyping of the CFHR3^*^A/B alleles was performed by Sanger sequencing of *CFHR3* exon 5 (24). Genetic analysis of the Hungarian patients was done as described previously (11).

### WBs Analyses of Plasma Samples

#### Primary and Secondary Antibodies

Rabbit polyclonal antibodies recognizing FH and different FHRs were generated in-house, or kindly provided by Dr. Richard Pouw and Dr. Mihály Jozsi. The anti-FH monoclonal antibodies (mAb) OX24 and C18 were from ThermoFisher (MA170057 and GAU0180302); the anti-FHR-1/FHR-2 mAb JHD7 was from Hycult Biotech (HM2301); the anti-FHR-4 mAb (MAB5980) and the anti-FHR-5 mAb (MAB3845) were from R&D. Rabbit polyclonal anti-human transferrin antibody PA527306 was from ThermoFisher. HRP-conjugated goat anti–rabbit IgG and HRP-conjugated goat anti–mouse IgG were obtained from Santa Cruz, and used as secondary antibodies. Primary and secondary antibodies were diluted in Tris/Tween buffer containing 2% ECL Advance blocking agent (GE Healthcare). Secondary antibodies solutions also contained the Streptactin reagent (Bio-Rad), to further visualize the molecular weight markers by chemiluminescence.

#### Western Blot Protocol

Plasma proteins (1-2 μL of EDTA-plasma) were separated on 10% polyacrylamide gels by SDS-PAGE under the following conditions: 50 mA/30 min; 75 mA/30 min; 100 mA/90 min. Molecular weight markers (WesternC blotting standards; Bio-Rad) were also loaded in every gel. Proteins were then transferred to nitrocellulose membranes (iBlot™ Transfer Stacks) using an iBlot Dry Blotting System (ThermoFisher), and blocked overnight at 4°C with 2% ECL Advance blocking agent in Tris/Tween. The membranes were incubated at room temperature with primary antibodies for 2 h, and with secondary antibodies for 30 min, and developed with a chemiluminescent substrate (ECL Advance Kit; GE Healthcare). Gel images were detected in a CCD camera (UVITEC Cambridge). Tris/Tween buffer was used for all washing steps.

Two-dimensional Western-blot analysis of FH/FHRs was done following our previously described protocol (25). Briefly, 200 μL of EDTA-plasma samples were adsorbed in heparin columns under low ionic strength, and 150 μg of the protein eluate (free from plasma albumin and immunoglobulins) were subjected to analytical Isoelectrofocusing (first dimension) using 7 cm-IPG strips of pH 3-10 or pH 4-7 (GE Healthcare). The IPG strips were then subjected to SDS-PAGE (second dimension) and Western-blot as described above.

### ELISA Assay for FHR-5 Levels

A sandwich ELISA that uses two capture antibodies and was originally developed by Dr. Elena Goicoechea de Jorge (Department of Immunology, Complutense University of Madrid) was adopted with small modifications. 96-well microtiter plates were coated with 50 μL of goat anti-mouse IgG_2a_ (Southern Biotech, 1080-01, 1/5,000 in PBS) and incubated overnight at 4°C. Plates were washed twice with washing buffer (PBS-0.2% Tween 20), and blocked for 1 h at 37°C with 100 μL of blocking buffer (PBS-1% BSA). After three washes, the plates were incubated for 1 h at 37°C with 50 μL of an in-house monoclonal antibody which recognizes FHR-1, FHR-2, and FHR-5 (2C6, IgG2a isotype, 1/4,000 dilution). Plates were washed four times, and 50 μL of 1/800 and 1/1,600 dilutions of plasma samples were incubated for 1 h at 37°C. After 4 washes, 50 μL of a mouse anti-FHR-5 monoclonal antibody (MAB3845 from R&D, IgG1_1_ isotype, 1/500 dilution) were added, and incubated at 37°C for 1 h. Plates were washed 4 times, incubated at 37°C for 30 min with peroxidase-conjugated goat anti-mouse IgG_1_ (1/5,000 dilution), and washed five times. A colored reaction was developed by using O-phenylenediamine dihydrochloride as substrate, the reaction was stopped with 10% sulfuric acid, and the absorbance was measured at 492 nm. A plasma sample with known FHR-5 levels was used as a standard curve.

### Neuraminidase Activity Assay

Neuraminidase activity in plasma samples was determined by using the Neuraminidase Activity AssayKit (MAK121; Sigma-Aldrich) following the manufacturer's protocol. Briefly, 20 μL of whole plasma or plasma dilutions (1/5, 1/10, 1/50) were loaded onto 96-well microtiter plates; upon addition of 80 μL of the reaction mix, the plates were incubated at 37°C for a total of 50 min, with absorbance readings at 570 nm at 20 min and 50 min. The absorbance increase from 20 to 50 min was used to calculate neuraminidase activity using a standard curve.

### FH Desialylation and Lectin Blotting

Twenty-five μg of FH (CompTech) in a volume of 25 μL were added to 100 μL of 0.1 M sodium acetate, pH 5, 25 μL of 1% BSA and 25 μL of *Clostridium perfringens* Neuraminidase (Sigma-Aldrich). Upon incubation at 37°C for 4 h under gentle shaking, the reaction was stopped with 25 μL of 0.5 M sodium hydrogen carbonate, pH 9.8. The same amount of FH was incubated in parallel in the same conditions, but without neuraminidase. The two FH samples (native and neuraminidase-treated) were loaded in triplicate on a 10% polyacrylamide gel, and after SDS-PAGE the samples were transferred to a nitrocellulose membrane as described above. The membrane was cut into 3 sections, each containing native and neuraminidase-treated FH; one section was incubated with rabbit polyclonal anti-FH antibodies, and the other two sections were incubated with two lectins with different sugar specificity, as described below.

Lectin RCA-I (*Ricinus Communis Agglutinin I*), which preferentially binds β-D-galactose residues, and lectin SNA (*Sambucus Nigra Agglutinin*), which binds α(2–6)-linked sialic acids, were purchased from Vector Laboratories, and used for blotting as reported (26). Membranes were blocked with MAL buffer (10 mM HEPES, pH 7.5, 150 mM NaCl, 0.2% BSA, 0.2% Tween-20) for 1 h at room temperature, and incubated overnight with 10 mL of 1 μg/mL biotinylated SNA or RCA-I in SNA buffer (10 mM HEPES, pH 7.5, 150 mM NaCl, 1% BSA, 0.1% Tween-20, 1 mM CaCl_2_, 1 mM MgCl_2_, 1 mM MnCl_2_). Membranes were washed three times for 10 min with 10 mL of SNA buffer, and then incubated for 1 h with 10 mL of 1 μg/mL streptavidin coupled with horseradish peroxidase (HRP). After 3 additional washes, the membranes were developed with a chemiluminescent substrate (ECL Advance Kit; GE Healthcare).

### Binding of FH and Desialylated FH (dFH) to C3b-Coated Microtiter Plates

The binding of FH and dFH to surface-bound C3b was determined according to our reported ELISA protocol (27), with a few modifications. 96-well polystyrene microtiter plates (Nunc MaxiSorp®) were coated overnight at 4°C with 0.4 μg of purified C3b in 100 μL of 0.1 M NaHCO_3_, pH 9.5. Plates were washed three times in TNT buffer (50 mM Tris/ HCl, pH 7.4, 150 mM NaCl, 0.2% Tween 20), and the wells were blocked at 37°C for 1 h with 1% BSA-TNT buffer. After washing, 100 μL of serial dilutions (from 2 μg/mL to 0.0325 μg/mL) of FH or dFH in 1% BSA-TNT buffer were added in duplicate, and allowed to interact with the surface-bound C3b at 37°C for 1 h. After three washes, 100 μL of an in-house rabbit anti-human FH polyclonal antibody which lacks reactivity against human C3b were added, and the plates were incubated at 37°C for 1 h. After three more washes, 100 μL of a 1/1,000 dilution of goat anti-rabbit immunoglobulin G antibody coupled with HRP (Santa Cruz) was added, and the plates were kept at 37°C for other 30 min. The plates were washed three times, and the enzymatic reaction was developed with ABTS (Merck), and stopped with 0.1% sodium azide. The binding of FH/dFH to the C3b-coated wells was determined by reading absorbance at 405 nm.

### Proteolytic Assays of C3b by FI

The cofactor activity of FH and dFH in the proteolytic cleavage of C3b by FI in the fluid phase was determined basically as described (28). Purified C3b (750 ng), FI (125 ng), and FH/dFH (100 ng) were diluted in 25 μL of 10 mM HEPES buffer, pH 7.5, 0.02% Tween 20 in Eppendorf microtubes (final concentrations: 170 nM C3b, 57 nM FI, 26 nM FH/dFH). Proteins were incubated at 37°C during 2.5 or 12.5 min, and after addition of 5 μL of 5X SDS-sample buffer solution with β-mercaptoethanol, 3 μL-aliquots were subjected to 10% SDS-PAGE and Western-blot, as described above. An anti-C3 antibody generated in rabbits (ab200999, Abcam) was used as a primary antibody; this antibody recognizes the α′chain of C3b and the α45 fragment of iC3b, but not the β chain. Upon completion of the Western-blot protocol, the gel images were analyzed with the ImageQuant TL software (GE Healthcare). For every incubation time, the intensity of the C3bα′ band plus the intensity of the iC3bα45 band in the gel lane was set to 100%, and the amount of C3b cleavage was then calculated as the percentage of the remaining C3bα′ band.

To analyse the cofactor activity of FH/dFH in the proteolytic cleavage of C3b by FI in the solid phase, 600 ng of C3b in 30 μL of PBS were added to microtiter wells, and incubated 1 h at 37°C. After 3 washing steps with PBS, 30 μL of a solution containing 100 ng of FI and 80 ng of FH/dFH were added (final concentrations: 100 nM C3b, 38 nM FI, 17 nM FH/dFH). Five μL of 5X SDS-sample buffer solution with β-mercaptoethanol were immediately added to one of the wells (0 time point). The plate was then incubated at 37°C, and the proteolytic reaction in the other wells was stopped after 2.5, 12.5, or 22.5 min by addition of 5 μL of 5X SDS-Sample buffer solution. The well content was carefully mixed by hand, and analyzed by 10% SDS-PAGE and Western-blot, using the same protocol as for the proteolysis in the fluid phase.

### ELISA Assay for C3 Convertase Decay-Accelerating Activity

The decay-accelerating activity of FH/dFH was analyzed by generating Properdin-stabilized C3bBb (C3bBbP) on microtiter plates. One hundred μL of 5 μg/mL C3b in PBS were immobilized overnight at 4°C on microtiter plates (Nunc Medisorb). Plates were washed three times with assay buffer (2.5 mM sodium barbitone, pH 7.4, 71 mM NaCl, 0.15% Tween, 1 mM MgCl_2_, 1 mM NiSO_4_) and blocked for 1 h at 37°C with 1% BSA-assay buffer. C3bBbP was then generated by adding 50 μL of a solution containing 2 μg/mL FB, 0.2 μg/mL FD, and 4 μg/mL Properdin, in 1% BSA-assay buffer. Increasing concentrations of FH/dFH (from 0.039 to 5 μg/mL) were then added, and incubated at 37 °C for 30 min. Plates were washed in assay buffer, and the remaining C3bBbP molecules were detected with a murine anti-Bb antibody (A227, Quidel; 1/500, 37°C, 1 h). After washing, a 1/2,500 dilution of a peroxidase-conjugated goat anti-mouse IgG (Jackson ImmunoResearch) was incubated for 1 h. A colored reaction was developed by using ABTS (Merck) as peroxidase substrate, and absorbance was read at 405 nm.

### Haemolytic Assays on Sheep Erythrocytes

Lysis of sheep erythrocytes by a serum sample from an aHUS patient carrying the FH mutation W1183L was performed as described (29). The amount of patient's serum giving about 50% lysis was then chosen to compare the capacity of increasing concentrations of FH/dFH (from 2.5 to 20 μg/mL) to prevent lysis.

Lysis of sheep erythrocytes by a normal human serum was induced by adding different amounts of the FH monoclonal antibodies OX24 (recognizing SCR5) or C18 (recognizing SCR20), as already reported (30). The amount of each antibody capable to induce 60–70% lysis was then used to test the capacity of increasing concentrations of FH/dFH (from 1 to 15 μg/mL) to prevent lysis.

In all the experiments, sheep erythrocyte lysis was calculated by reading absorbance at 414 nm.

## Results

### Complement Rare Variants and Risk Polymorphisms in the Spanish Cohort of SP-HUS

From 2006 to 2019 we performed complement studies in 13 Spanish HUS patients (seven males and six females) who were diagnosed in the context of an *S. pneumoniae* infection (Table 1). All the patients but one were younger than 3 years at disease onset. Genetic screening of *CFH, MCP, CFI, CFB, C3*, the five *CFHR* genes, and other complement genes was undertaken in nine patients; genetic screening could not be done in one patient, and was uncompleted in three patients.

**Table 1:** Complement findings in the Spanish SP-HUS cohort.

**Patient code**	**Gender**	**Age at onset**	**Rare Variants**	**Common aHUS-risk variants**				***DelCFHR3-CFHR1***	**C3/C4 (mg/dL)**
				***MCPggaac***	***CFH(H3)***	CFHR3*B	CFHR1*B		
H118	Female	2 y	No	No	HET	HET	HOM	No	178/27.2
H150^a^	Male	12 mo	*CFHR3 (c.796+1G>A)*	No	No	No	HET	HET	132/34
H171^a,b^	Female	3 y	No	No	HET	HET	HET	No	81.3/40.6
H201	Female	19 mo	Uncomplete screening	Not done	No	HET	HOM	No	111/42.3
H202	Female	2 y	Uncomplete screening	Not done	No	No	HET	No	145/33.1
H582	Female	47 y	No	No	HET	HET	No	No	150/46.1
H619^c^	Male	2 y	CFHR5 (c.486_487insAA; p.E163Kfs*10)	HET	No	No	HET	No	166/44.8
H640	Male	5 mo	*CFI (c.1534+5G>T)*	No	No	No	No	No	153/30.3
H678	Male	12 mo	Uncomplete screening	Not done	HET	HET	HOM	No	123/26.2
H731	Female	17 mo	*CFHR5 (c.368A>G; p.N123S / c.832G>A; p.G278S)*	HET	HET	HOM	HET	No	143/36
H837	Male	21 mo	No	No	No	No	HET	No	87.1/9.6
H859	Male	16 mo	*C1QB (c.223G>A p.G75R)*	No	HET	HET	HOM	No	146/60
H946	Male	21 mo	Genetic screening not done	Not done	HET	HET	HOM	No	58.9/9.56

Demographic and complement data of the 13 SP-HUS patients of Spanish origin studied during 2006–2019. Genetic data include rare complement variants, the aHUS-risk haplotypes MCPggaac, CFH(H3), CFHR3*B, and CFHR1*B, and the common DelCFHR3-CFHR1 variant. C3 and C4 levels in the first plasma sample available are also shown; normal ranges were 75-135 mg/dL for C3, and 14-60 mg/dL for C4.

a *Described in (25)*.

b *This patient presented anti-FH autoantibodies*.

c *Described in (12)*.

Rare complement variants were found in heterozygosis in five patients (four males and one female). Patient H150 carries a rare *CFHR3* variant (c.796+1G>A) that alters normal splicing and results in a null allele, and he also carries the *CFHR3-CFHR1* deletion (*DelCFHR3-CFHR1* or Δ_*CFHR*3−*CFHR*1_); thus, the two variants generate homozygous FHR-3 deficiency in this patient (25). Patient H619 presents partial FHR-5 deficiency, and he has recently been described together with a glomerulonephritis patient carrying a very similar variant (12). The *CFI* intronic variant (c.1534+5G>T) found in patient H640 is located within the donor splicing site of exon 11, but the patient had normal FI levels. The *CFHR5* variant in patient H731 (c.368A>G; p. Asn123Ser) was predicted to be likely benign; nonetheless, this patient also carries another *CFHR5* variant (c.832G>A; p.Gly278Ser) that is a null allele, generating FHR-5 haploinsufficiency in the patient. Finally, patient H859 carries a genetic variant in the *C1QB* gene (c.223G>A; p.Gly75Arg) that results in an amino acid change at position 75 of the C1qB chain; C1q levels in this patient were normal.

Analysis of the *MCPggaac* and *CFH(H3)* aHUS-risk haplotypes, and of the aHUS-risk alleles CFHR3^*^B and CFHR1^*^B, could be done in most patients. Two out of nine patients (22%) were carriers of the *MCPggaac* risk haplotype, while the *CFH(H3)* risk haplotype was found in 7 out of 13 patients (54%), six of whom also carried the CFHR3^*^B and CFHR1^*^B alleles. We then compared the frequency of these variants in the 13 SP-HUS patients with the frequencies observed in 22 pregnancy-associated HUS patients (P-aHUS) (31), 352 patients from our aHUS cohort (24), and a total of 227 Spanish control individuals. As it is shown in Table 2, the *MCPggaac* risk haplotype is less frequent in the SP-HUS cohort, while the frequencies of the CFHR1^*^B and CFHR3^*^B risk alleles are higher than in controls, and comparable to the frequencies observed in the aHUS and P-aHUS cohorts.

**Table 2:** Contribution of aHUS-risk haplotypes to SP-HUS.

**Haplotype**		**Control**	**aHUS**	**SP-HUS**	**P-aHUS**
*MCPggaac*	N	222^a^	662	18	44
	Frequency	0.333	0.414	0.111	0.432
*CFH(H3)*	N	186^a^	668	26	44
	Frequency	0.242	0.302	0.269	0.455
CFHR3*B	N	194^b^	634	26	26
	Frequency	0.242	0.355	0.346	0.462
CFHR1*B	N	204^b^	610	26	44
	Frequency	0.368	0.467	0.615	0.455

Frequency of the aHUS-risk haplotypes MCPggaac, CFH(H3), CFHR3*B and CFHR1*B in the Spanish cohorts of aHUS (352 patients), SP-HUS (13 patients), and P-aHUS (22 patients), and in a total of 227 Spanish control individuals.

a *Frequencies of MCPggaac and CFH(H3) were determined in one cohort of 116 control individuals*.

b Frequencies of CFHR3*B and CFHR1*B were determined in a cohort of 111 control individuals. N represents the number of chromosomes analyzed.

### Complement Studies in Plasma Samples From Spanish and Hungarian Patients

We determined the complement profile (i.e., levels of C3, C4, FH, FI, and anti-FH autoantibodies) in plasma samples from all the Spanish SP-HUS patients. Most plasma samples were obtained between 1 month and 3 years after disease onset, and they had normal C3 and C4 levels. Low C3 and C4 levels, revealing complement activation by the classical pathway, were only detected in patient H946, and, to a lesser extent, in patient H837 (Table 1). Because these two plasma samples were obtained in the first week after HUS onset, it could not be excluded that complement activation had also happened in the other patients during active infection. Anti-FH autoantibodies were only detected in patient H171, who does not carry the *CFHR3-CFHR1* deletion.

**Figure 1:**
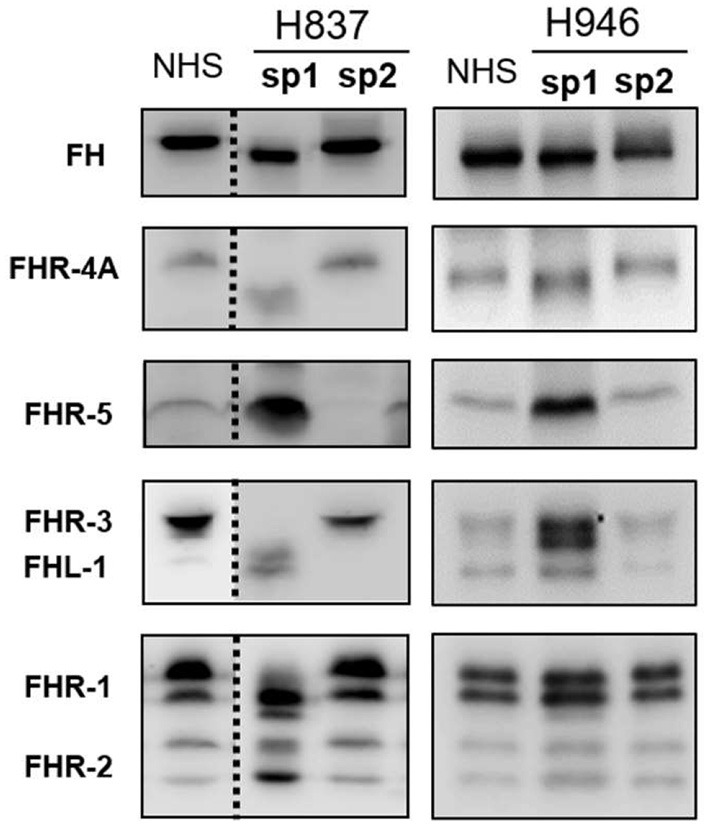
1D-WB analysis of FH/FHRs in Spanish patients H837 and H946. Two plasma samples from patients H837 and H946 drawn during the acute phase (sp1) or at remission (sp2) were analyzed by WB with polyclonal and monoclonal antibodies against FH and the FHR proteins. Samples from patient H837 were obtained 1 day (sp1) and 3 months (sp2) after SP-diagnosis. Samples from patient H946 were obtained 4 days (sp1) and 2 months (sp2) after SP-diagnosis. A plasma sample from a control individual was included in the same gel; the dotted lines denote that the control sample and the samples from patient H837 were not consecutive.

**Figure 2:**
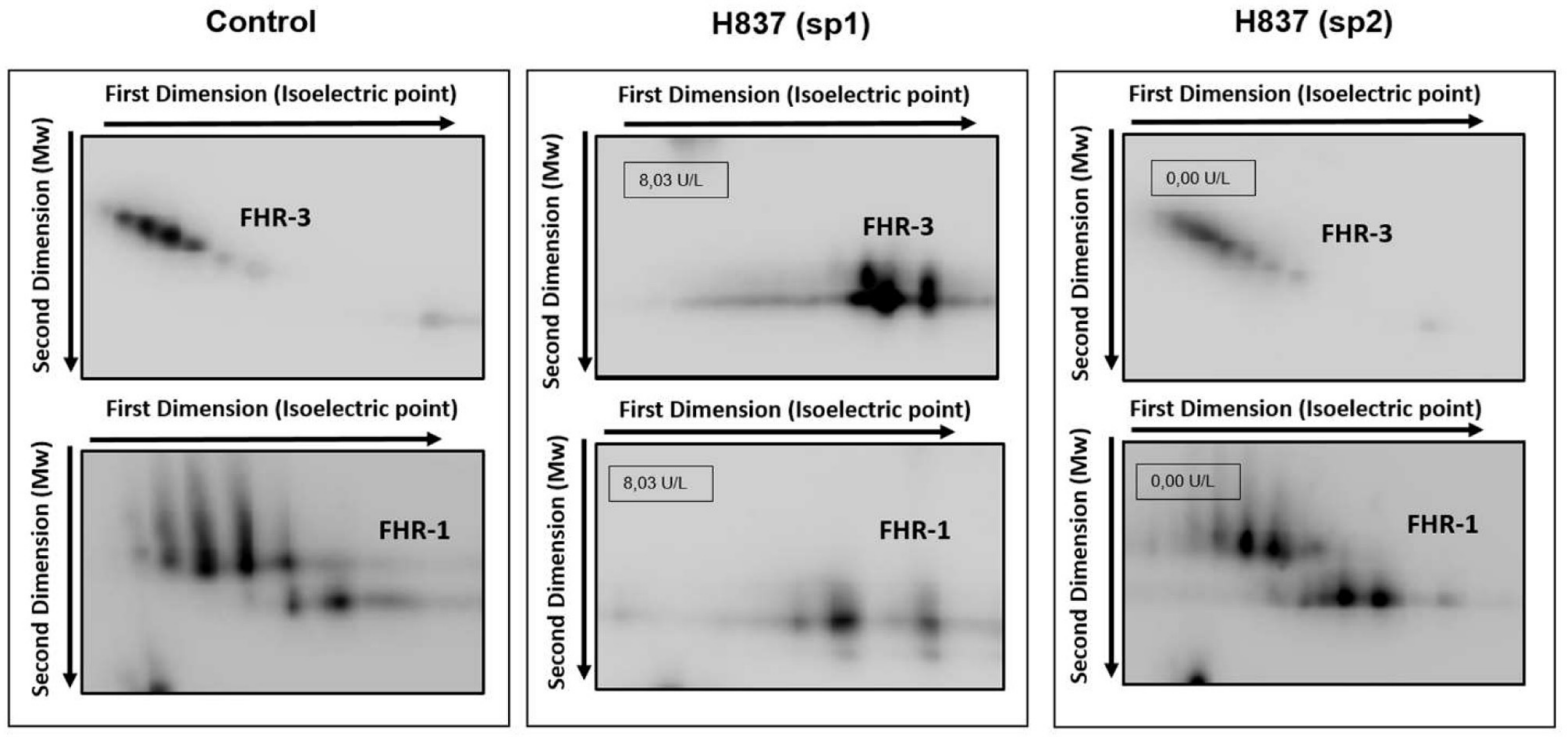
Desialylation of FHR-1 and FHR-3 in patient H837 by 2D-WB analysis. FHR1 and FHR3 partially purified from sp1 and sp2 from patient H837 and from a control individual were subjected to 2D-electrophoresis and Western-blot with polyclonal antibodies. The characteristic ladder of FHR-3 spots (each having different Mw and isoelectric point) appeared as a few basic spots of lower Mw in the patient sample obtained 1 day after SP-HUS diagnosis (H837 sp1), and was recovered in the sample drawn 3 months later (H837 sp2). A similar situation applies to FHR-1. The small boxes within the gel images indicate the neuraminidase activity detected in samples sp1 and sp2 from patient H837.

All plasma samples were also analyzed by Western-blot with different sets of polyclonal and monoclonal antibodies recognizing FH and the FHRs. These analyses revealed that FH and the FHRs proteins in the plasma samples from patients H837 and H946, drawn at disease onset, presented a lower Molecular weight (Mw) than the control sample. We could analyse a second plasma sample from these patients, obtained at disease remission, and we observed that the Mw of FH and FHRs was normal (Figure 1). These findings suggested desialylation of FH/FHRs in patients H837 and H946 by the pneumococcal neuraminidase(s). Because sialic acid removal would also decrease the negative charge of the protein and increase its isoelectric point (pI), we performed 2D-Western blot analysis of plasma samples from patient H837 drawn at onset and at remission, following our reported protocol (25); in this kind of analysis, proteins are first separated according to their pI, and then according to its Mw. As it is shown in Figure 2, the characteristic 2D-pattern of FHR-3 and FHR-1 isoforms is drastically altered in the onset sample: there are fewer isoforms, with lower Mw and higher pI than in a control sample, or than in the remission sample. We interpreted this finding as a confirmation of transient desialylation of plasma glycoproteins by the pneumococcus. We then quantified neuraminidase activity in the two samples from patient H837 and in a control sample with an “*in vitro*” assay; a high neuraminidase activity (8.03 U/L) was detected in the onset sample, while no enzymatic activity was observed in the remission sample (0.01 U/L) and in the control sample (0.02 U/L). Neuraminidase activity (0.45 U/L) was also detected in the onset sample from patient H946, but not in the remission sample (0.01 U/L).

**Figure 3:**
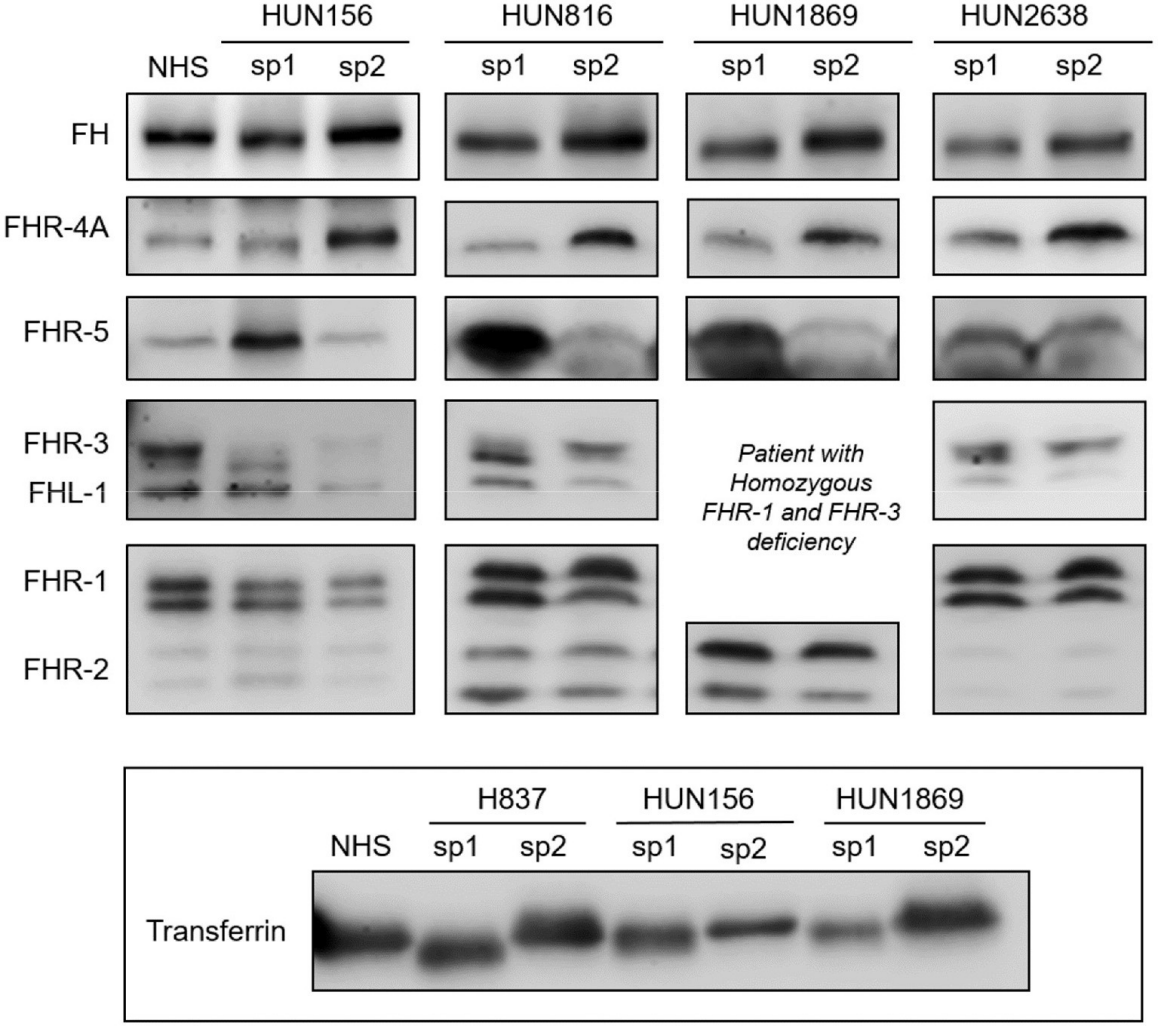
Desialylated FH/FHRs in samples from Hungarian SP-HUS patients. WB analyses of FH/FHRs in two plasma samples (sp1 and sp2) from four Hungarian patients. Time between disease onset and extraction date was as follows: HUN156 (10 days and 11 months); HUN816 (9 days and 2 months); HUN1869 (5 days and 2 months), this patient is homozygous for FHR-3 and FHR-1 deficiency; HUN2638 (23 days and 43 days). WB analysis of human Transferrin (Mw 77 kDa) in sp1 and sp2 samples from patients H837, HUN156, and HUN 1869 is shown at the bottom inset.

To determine whether the desialylation of FH/FHRs that we have observed in patients H837 and H946 was a general phenomenon in SP-HUS, we performed WB analyses in plasma samples drawn during the acute phase from 11 Hungarian SP-HUS patients. Patterns suggestive of desialylation (i.e., a lower Mw of FH and FHRs) were observed in four patients (Figure 3). WB analyses also revealed that the intensity of the FHR-5 band was higher in the samples drawn at disease onset than at remission; this was particularly evident for patient H837 (Figure 1) and patients HUN816 and HUN1869 (Figure 3). These differences were further confirmed by determining FHR-5 levels by ELISA (Table 3). All the samples showing FH/FHRs desialylation were also analyzed by WB with polyclonal antibodies recognizing human transferrin, a 77 kDa plasma glycoprotein; as it could be expected, the Mw of transferrin in those samples was lower than in the control sample (Figure 3), thus suggesting general desialylation of plasma glycoproteins by the pneumococcal neuraminidase.

**Table 3:** Transient desialylation of FH/FHRs in two Spanish and four Hungarian SP-HUS patients.

**Patient code**	**Gender**	**Age at onset (mo)**	**Previous vaccination**	**Time after onset**	**Clinical status**	**Complement profile**	**FHR-5 Levels^a^ (μg/mL)**	**FH/FHRs desialylation**	**Neuraminidase Activity (U/L)^b^**	**Genetic findings^c^**
H837	Male	21	No info available	1 days	Onset	Low C4, FH, FI	3.61	YES	8.03	No pathogenic variants
				3 mo	Remission	Normal	0.03	NO	0	
H946	Male	21	Prevenar	4 days	Onset	Low C3, C4	1.97	YES	0.45	Non-available
				2 mo	Remission	Normal	0.80	NO	0	
HUN156^d^	Female	18	Pneumovax	10 days	Onset	Low C3,C4,FB,FI	1.51	YES	3.94	*CFI* (c.148C>G, p.Pro50Ala); *MCPggaac*
				11 mo	Remission	Low FI	0.48	NO	0	
HUN816	Male	36	No info available	9 days	Onset	Low C3, C4, FH	2.81	YES	0.54	Non available
				2 mo	Remission	High C3, FI, FB	0.86	NO	0.47	
HUN1869	Female	30	No info available	5 days	Onset	Low C3, C4, FI	1.19	YES	2.50	*DelCFHR3-CFHR1* (HOM)
				2 mo	Remission	Normal	0.42	NO	0.17	
HUN2638	Male	32	Prevenar	23 days	Onset	Low C3, C4, FH	1.08	YES	0.42	*C3 (c.2852G>A, p.Arg951His)*; *C3 (c.304C>G;R102G*); C3 P314L; *CFH (c.184G>A; V62I)*; *CFH(H3); MCPggaac*
				42 days	Remission	Normal	0.86	NO	0.44	

*Complement profile and neuraminidase activity in the two plasma samples drawn during disease onset and at remission. Anti-FH autoantibodies were negative in all samples*.

a *Mean levels in controls: 1.98 ± 1.02 μg/mL*.

b *Background level in controls: 0.50 U/L*.

c *All genetic variants in patients HUN156 and HUN2638 are in heterozygosis*.

d *Described in (11)*.

A summary of demographic data, complement findings and neuraminidase activity in the samples from the six patients (two Spanish and four Hungarian) with transient desialylation is depicted in Table 3. Acute phase samples were drawn between 1 and 23 days after diagnosis, and they showed low C3 and C4 levels but no anti-FH antibodies. Remission samples were drawn between 42 days and 11 months after diagnosis, and presented normal C3 and C4 levels. Neuraminidase activity ranged from 0.17 U/L (remission sample from patient HUN1869) to 8.03 U/L (acute sample from patient H837). Nonetheless, a clear correlation between neuraminidase activity, days after diagnosis and desialylation was not observed in all the patients. Complement genetic variants were observed in three Hungarian patients. Patient HUN156 has a rare variant in *CFI* (c.148C>G, p.Pro50Ala; (11)); patient HUN2638 has a rare variant in *C3* (c.2852G>A, p.Arg951His), and patient HUN1869 presents homozygous FHR-3 and FHR-1 deficiency.

### Functional Relevance of FH Desialylation

**Figure 4:**
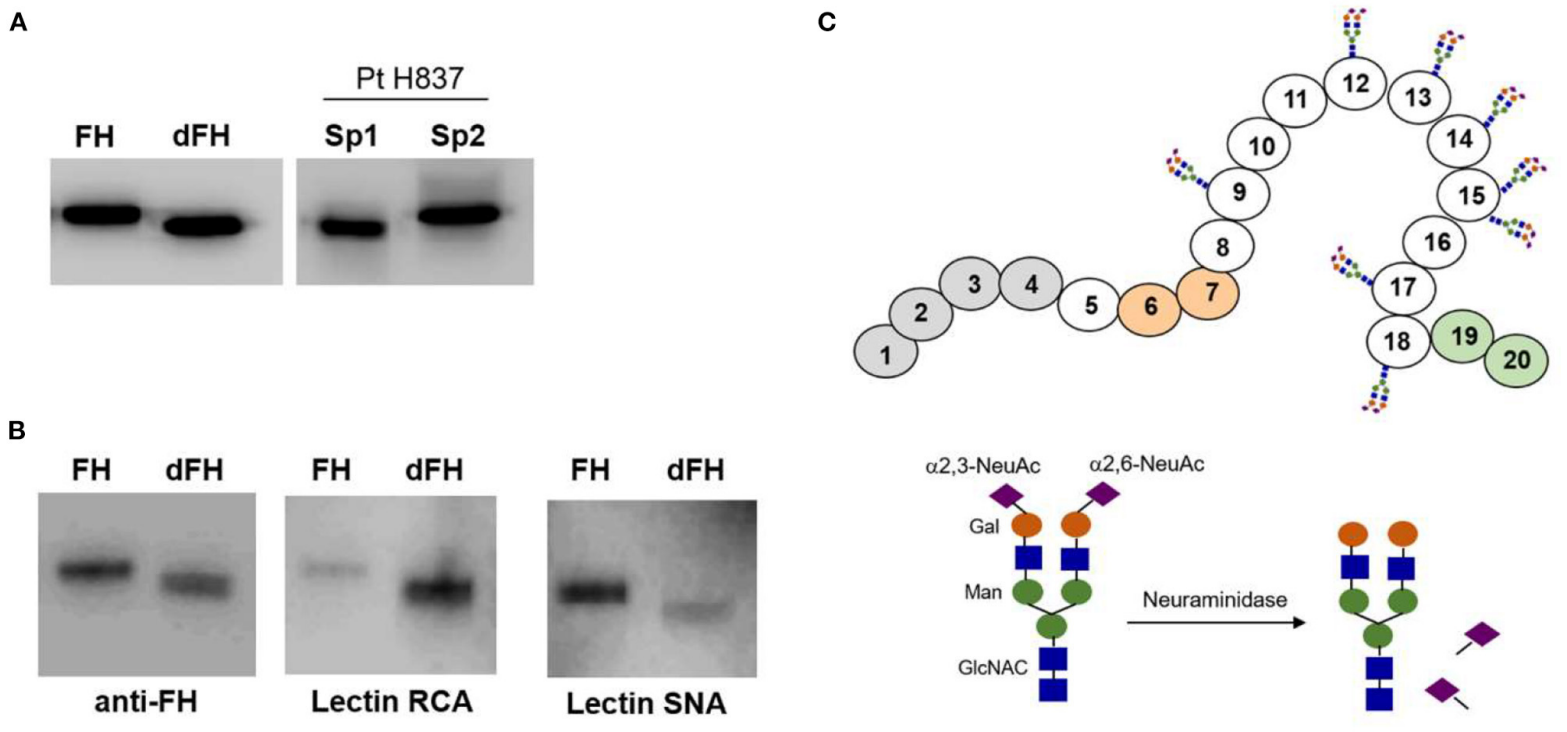
Lectin recognition of FH and desialylated FH. **(A)** Western-blot analysis with polyclonal anti-FH antibodies. “*In vitro*” desialylated FH (dFH) has the same molecular weight than FH in a plasma sample from patient H837 obtained 1 day after disease onset (sp1), and lower than FH in the sample obtained at remission (sp2). **(B)** Differential recognition of FH and dFH by lectin RNA (which recognizes non-reducing terminal beta-D-galactose), and lectin SNA (which binds primarily to Neu5Ac(α2–6) Gal/GalNAc disaccharide sequences). **(C)** Schematics of FH showing the SCR domains with N-linked carbohydrates; the sugar composition of the carbohydrate molecule is based on Schmidt et al. (32).

We wanted to know whether desialylation altered the functional activity of FH, but we could not purify it from any of the patients' samples drawn at disease onset because of limited sample volume. Therefore, to approach the potential relevance of FH desialylation, we generated dFH “*in vitro*” from commercially available FH, purified from human plasma. Figure 4A shows that dFH has the same Mw than FH in the onset sample from patient H837. We also checked sialic acid removal from purified FH by analyzing the binding of lectins RCA-I and SNA by Western-blot (Figure 4B). Lectin SNA binds predominantly to α(2–6)-linked sialic acids (present in FH but absent in dFH), while lectin RCA-I binds predominantly to β-galactose residues, which become fully accessible after desialylation. Thus, the preferential binding of lectin SNA to FH, and of lectin RCA-I to dFH confirmed “*in vitro*” FH desialylation.

**Figure 5:**
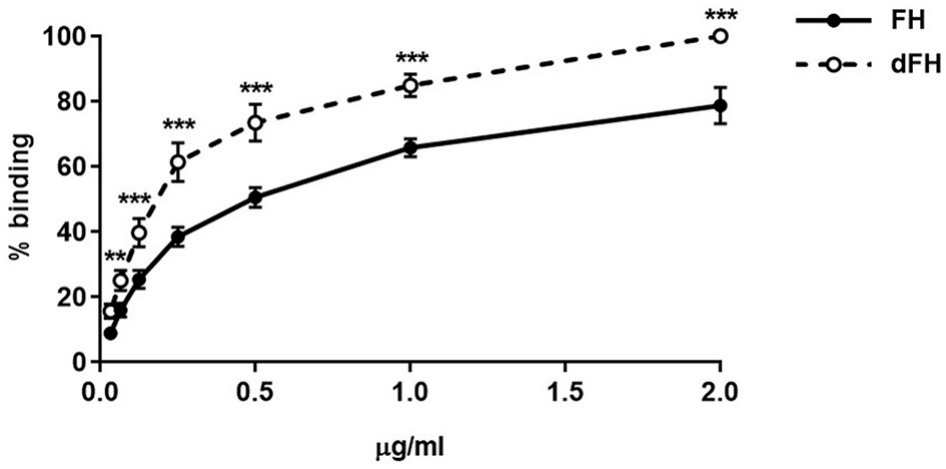
Binding of FH and dFH to surface-bound C3b. Binding of increasing concentrations of FH and dFH to microtitre plates coated with 400 ng of C3b. Upon addition of a polyclonal antibody which recognizes FH and dFH, absorbance was read at 420 nm, and the maximum binding observed was set as 100%. The two curves represent the mean ± SD from four independent ELISA experiments. **P < 0.01; ***P < 0.001.

**Figure 6:**
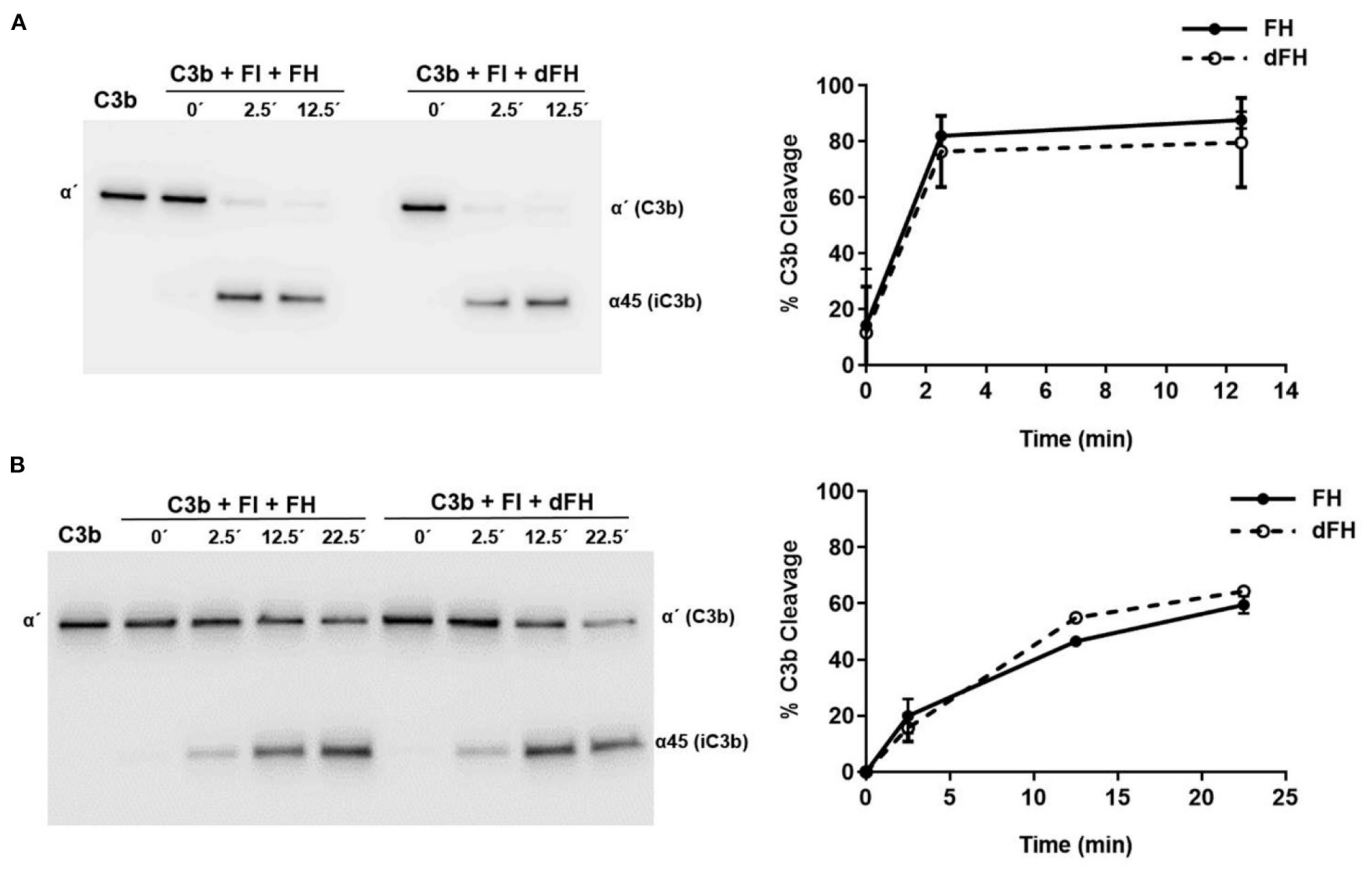
Cofactor activity of FH and dFH in the proteolysis of C3b by Factor I. Proteolysis of C3b by FI in the fluid phase **(A)** or on microtiter plates **(B)**, by using FH or dFH as cofactors. The proteolytic activity at 2.5, 12.5, and 25 min was analyzed by Western-blot with an antibody which recognizes the α′ chain of C3b (substrate), and the α45 fragment of iC3b (product). Proteolytic cleavage was expressed as the relative intensity of the remaining C3bα′ chain at each time point. All curves represent the mean ± SD from three independent experiments.

**Figure 7:**
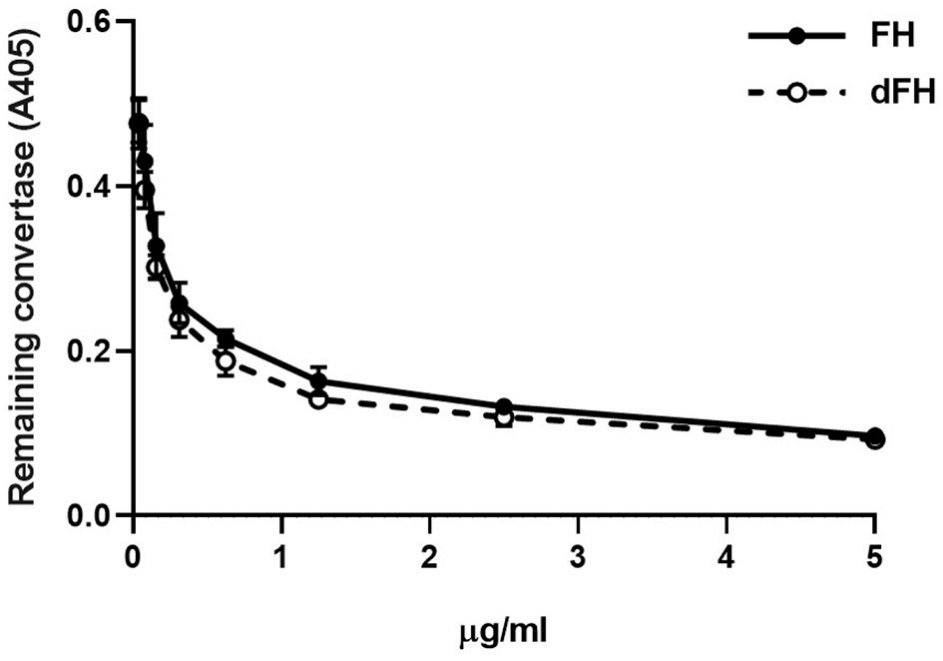
Decay-accelerating activity of FH and dFH on surface-bound C3bBb(P) convertase. The alternative pathway C3bBbP convertase was built up on microtiter plates. After addition of increasing concentrations of FH or dFH, the remaining C3bBbP molecules were detected with a monoclonal anti-factor Bb antibody. Data are the mean ± SD from three independent ELISA experiments.

We then compared the functional activity of FH and dFH in different experimental settings. We used an ELISA assay to analyse the binding of FH/dFH to surface-bound C3b, and observed that dFH bound more efficiently than native FH (Figure 5), suggesting that sialic acid removal favors the interaction of FH with surface-bound C3b. The higher binding of dFH, nonetheless, did not increase the cofactor activity of FH and dFH, either in the fluid phase or on surfaces. As illustrated in Figure 6, the cleavage of soluble or surface-bound C3b by Factor I to generate iC3b was not affected by using FH or dFH as cofactors. In the same way, no differences were observed when comparing the ability to dissociate solid-phase, preformed C3bBb(P) convertase, as similar decay-accelerating activities were observed with FH and with dFH (Figure 7).

**Figure 8:**
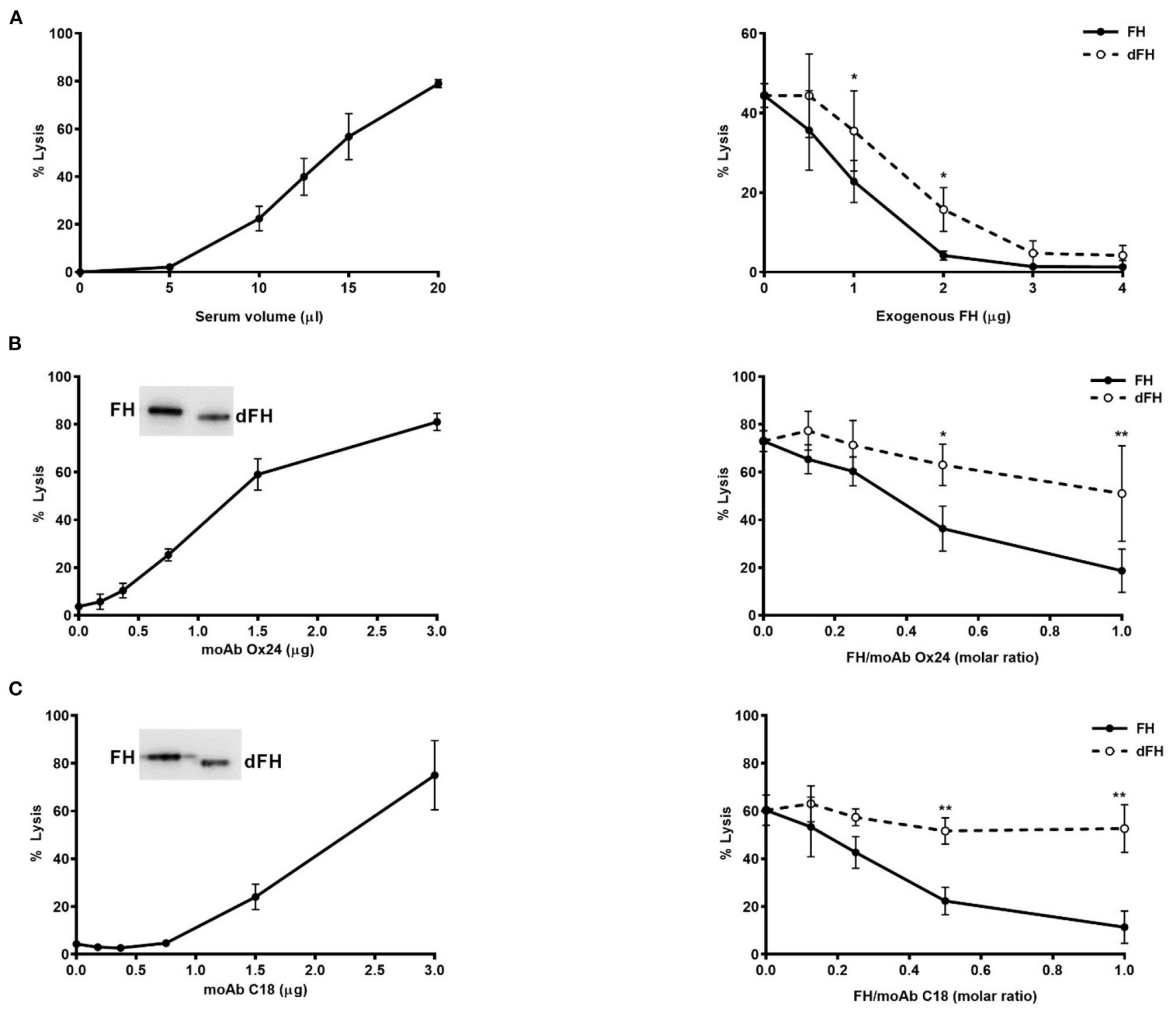
Haemolytic assays on sheep erythrocytes. **(A)** Lysis of sheep erythrocytes by the serum from an aHUS patient carrying the mutation W1183L in FH (left panel). The lysis generated by 12.5 μL of patient's serum (about 50%) could be prevented by addition of exogenous FH or dFH, but dFH was less effective (right panel). **(B,C)** Lysis of sheep erythrocytes was induced by adding increasing amounts of monoclonal antibodies OX24 **(B)** or C18 **(C)** to 20 μL of NHS (left panels). MoAb OX24 recognizes FH SCR4 domain, and MoAb C18 recognizes FH SCR20 domain; both antibodies recognize similarly FH and dFH by Western-blot (insets). The lysis induced by 1.6 μg of OX24 (about 70%) or C18 (about 60%) could be inhibited by adding increasing concentrations of FH or dFH, although dFH was less effective (right panels). All curves represent the mean ± SD from three independent experiments. *P < 0.05; **P < 0.01.

To analyse the whole effect of FH desialylation on complement regulation on cellular surfaces, we used two different formats of haemolytic assays on sheep erythrocytes. The first format is our original assay of sheep erythrocyte lysis by the serum of an aHUS patient who carries the FH mutation W1183L (29). The second format is a modification of this assay, in which the addition of anti-FH monoclonal antibodies OX24 (targetting FH SCR4) or C18 (targeting FH SCR20) to a NHS renders it capable to lyse sheep erythrocytes (30). In both formats of haemolytic assays, we compared the capacity of exogenous FH/dFH to prevent haemolysis. Interestingly, we observed that FH desialylation clearly decreased its ability to prevent sheep erythrocytes lysis (Figure 8), suggesting the contribution of FH's own sialic acids on its regulatory activity on cellular surfaces.

## Discussion

An infrequent complication of *S. Pneumoniae* infections is the Haemolytic Uraemic Syndrome (HUS), a clinical entity characterized by the triad of thrombocytopenia, microangiopatic haemolytic anemia and acute renal failure (33). The contribution of complement pathogenic variants and risk polymorphisms in the atypical forms of HUS is very well-established, and screening of the complement genes *CFH, MCP, CFI, CFB, C3*, and *CFHRs* in these patients is mandatory (10). Complement studies in HUS associated to *S. Pneumoniae* (SP-HUS), however, are very limited because it has generally been considered that this is a secondary manifestation of the infection process (34).

We have performed complement genetic screening in 9 Spanish SP-HUS patients, and observed that five of them carry a total of six rare genetic variants. Three variants were null alleles in *CFHR3* (patient H150) or *CFHR5* (patient H619 and patient H731). FHR-3 competes FH binding to *Neisseria meningitidis* (35), thus decreasing bacterial survival. Although it is not known whether FHR-3 can also compete FH binding to *S. pneumoniae*, the lack of FHR-3 could be advantageous for the pneumococcus. This is difficult to determine, because isolated deficiencies of FHR-3 are very rare. Nonetheless, the combined deficiency of FHR-3 and FHR-1 as a consequence of the homozygous *DelCFHR3-CFHR1* deletion is relatively frequent, and there is no evidence that it predisposes to infections. The FHR-5 haploinsuficiency observed in patient H169 could decrease complement activation and increase infection susceptibility, although the clinical phenotype probably relies on additional, currently unknown risk factors (12).

The pathogenic relevance of the three other variants found in our SP-HUS patients is unknown. The *CFHR5* variant in patient H731 (c.368A>G; p.Asn123Ser), which abolishes one of the potential N-glycosilation sites in FHR-5, was predicted to be likely benign. The *C1QB* variant in patient H859 (c.223G>A; p. Gly75Arg) was reported in one individual with very early onset inflammatory bowel disease, and predictive tools suggested that it may alter the protein function (36). Thus, it is possible that this C1q variant has decreased capacity to activate the classical pathway and eliminate the pathogen, and/or that it binds the pneumococcal protein PepO with higher affinity, increasing bacterial adherence to the host's cells (37). The *CFI* intronic variant in patient H640 (c.1534+5G>T; rs114013791) has been described in an Italian aHUS patient (38), and in several patients from the Newcastle aHUS cohort (39), all of them having normal FI levels; the contribution of this variant, present in 1.55% of European controls, to the genetic predisposition to aHUS is thus uncertain. In conclusion, a significant proportion of the Spanish SP-HUS patients (five out of nine) carry rare genetic variants in complement genes, but their relevance to HUS predisposition is unknown.

The small sample size of our SP-HUS cohort (13 patients) does not allow to achieve statistically significant conclusions when comparing the frequency of the common genetic variants MCPggaac, CFH(H3), CFHR3^*^B, and CFHR1^*^B with control individuals, or with the aHUS cohort (Table 2). Nonetheless, our analyses reveal that the *MCPggaac* haplotype is underrepresented in the SP-HUS patients, where it has a lower frequency than in our cohort of 352 aHUS patients (0.111 vs. 0.414), or that in 22 P-aHUS cases (0.111 vs. 0.432). The relevance of this observation would require analyses in more SP-HUS patients, but it suggests that the membrane regulator MCP is not an important player in SP-HUS pathogenesis. It is also interesting that the frequencies of the aHUS-risk variants CFHR3^*^B and CFHR1^*^B in our SP-HUS cohort (0.346 and 0.615, respectively) are higher than in control individuals (0.242 and 0.368), and comparable to the frequencies observed in the aHUS (0.355 and 0.467) and P-aHUS (0.462 and 0.455) cohorts. These findings suggest that CFHR3^*^B and CFHR1^*^B are predisposing factors to SP-HUS. These two variants frequently segregate in an extended CFH(H3)-CFHR3^*^B-CFHR1^*^B haplotype that associates with reduced FH levels and increased FHR-3 levels (24, 40), but whether a local imbalance of the FH/FHR-3 ratio predisposes to SP-HUS will require further investigation.

The contribution of FH and FHR proteins to the pathogenic mechanism of SP-HUS could also result from the transient removal of their sialic acids by the pneumococcal neuraminidase. It has been observed that the sequential action of pneumococcal neuraminidase, galactosidase, and NAcglucosidase reduce complement deposition on the pathogen surface and its subsequent phagocytosis by human neutrophils, but the complement glycoprotein(s) affected are unknown (41). FH is the complement protein with more N-Glycosylation sites (nine sites), followed by C2 (eight sites), and FI (six sites). FH deglycosylation decreases its Mw by 17.9 kDa, and eight of its nine N-glycosylation sites are occupied by complex, diantennary sialylated, non-fucosylated glycans, although a few triantennary structures are also present (42). Most FH sialic acids are alfa2-6-linked to the carbohydrate chains (32), but the functional consequences of FH desialylation are not fully understood. We here show desialylation of FH and FHRs in plasma samples from 6 SP-HUS patients (two from Spain and four from Hungary), that we attribute to the activity of the pneumococcal neuraminidase on human glycoproteins (Figure 4). Because FH and FHRs desialylation was most evident in a plasma sample drawn only 1 day after disease onset, we believe that this is a general finding that disappears upon infection resolution. We also think that the desialylation process is independent of the presence of rare complement genetic variants; in fact, it was more evident in patient H837, who does not carry any pathogenic variant.

To determine whether sialic acid removal had any consequences on FH function, we compared the regulatory activity of native and “*in vitro*” desialylated FH by using assays in the fluid phase and on surfaces, which we had already used to check FH mutants purified from aHUS patients (27). Sialic acid removal increased FH binding to C3b-coated microtiter plates (Figure 5). This result agrees with the enhanced binding of a partially deglycosylated and desialylated recombinant FH molecule in biosensor experiments (43).

FH desialylation, nonetheless, did not affect its capacity to act as a cofactor of FI in the proteolysis of C3b in the fluid phase or on surfaces, as no differences between native and desialylated FH were appreciated (Figure 6). The same observation was reported by Schmidt et al. (43), who analyzed cofactor activity in the fluid phase, and did not find differences between plasma FH and the partially deglycosylated and desialylated recombinant FH. In line with these results, the partial deglycosylation of FI to remove sialic acids and Galactose residues did not affect the proteolysis of C3(NH_3_) (a structural C3b analogous) in the fluid phase (44). We conclude that in the proteolytic cleavage of C3b to iC3b, the sialic acid molecules of the enzyme (FI) or the cofactor (FH) do not play any relevant role.

We also observed that desialylated FH kept intact its capacity to dissociate preformed C3bBb(P) convertase (Figure 6). This result differs from the increased decay observed with the recombinant FH in biosensor experiments (43), and from the higher capacity of deglycosylated FH to dissociate properdin-stabilized C3bBb convertase preformed on the erythrocyte surface (45). These discrepancies could be due to the deglycosylation treatments of the FH molecules used in these previous reports, while we have only removed FH sialic acids, leaving the other sugar residues in the native carbohydrate molecules unchanged.

Because our functional assays on microtiter plates did not take into account the relevance of surface polyanions for the FH regulatory activity, we performed two different kind of haemolytic assays with sheep erythrocytes, which have polyanionic molecules on their surface. In the first assay, sheep erythrocytes are “spontaneously” lysed by the serum from an aHUS patient whose mutated FH cannot bind to the sheep erythrocyte surface and protect them from complement attack (29). In the second assay, the addition of specific anti-FH monoclonal antibodies to a normal human serum abolishes FH binding to the sheep erythrocyte surface, rendering them susceptible to complement-mediated lysis (30). When we compared the capacity of exogenous FH and desialylated FH to prevent sheep erythrocytes lysis, we observed a lower activity of desialylated FH in the two kind of assays (Figure 8), suggesting that FH desialylation decreases its capacity to regulate complement activation on the erythrocyte surface. As the other functional assays do not suggest any role for FH sialic acids on C3 convertase dissociation or in the proteolytic cleavage of C3b, we think that FH desialylation could somehow alter its interaction with polyanionic molecules on the cellular surface, and that this could result in decreased binding of desialylated FH to the cellular surface and decreased complement regulation. Further studies are required to determine the exact mechanism, and whether this transient dysregulation has a relevant role on SP-HUS pathogenesis, as already suggested (9, 46).

In summary, we here show that rare complement genetic variants in SP-HUS patients are more frequent than it could be expected, and that aHUS-risk polymorphisms in the *CFH-CFHR3-CFHR1* region likely contribute to SP-HUS. Based on these findings, we recommend complement genetic screening in patients who develop HUS in the context of *S. pneumoniae* infections, as well as to analyse aHUS-risk variants in these patients. We also show desialylation of human FH and FHR proteins by the pneumococcal neuraminidase at SP-HUS onset, and provide functional evidence suggesting that desialylated FH has a lower capacity to regulate complement activation on cellular surfaces.

## Data Availability Statement

The genetic datasets presented in this article are not readily available due to ethical restrictions of research participants. Requests to access experimental datasets should be directed to PS-C, pilar.sanchez-corral@idipaz.es.

## Ethics Statement

The studies involving human participants were reviewed and approved by Ethical Committees from La Paz University Hospital. Madrid, Spain, and from Semmelweis University. Budapest, Hungary. Written informed consent to participate in this study was provided by the participants' legal guardian/next of kin.

## Author Contributions

IG and FC performed Western-blots and functional studies, analyzed data, and prepared figures. PN was responsible for the analysis of the complement profile in plasma samples from Spanish patients. EA collected biological samples and was responsible for complement genetic screening from Spanish aHUS patients. AM, MM, and JB gathered clinical data. NV and ZP collected plasma samples and clinical and complement data from Hungarian patients. DC and ÁS performed genetic screening of Hungarian patients. PS-C designed the study, analyzed data, prepared figures and wrote the first draft of the manuscript. All the authors revised the data and contributed to the final version of the manuscript.

## Conflict of Interest

The authors declare that the research was conducted in the absence of any commercial or financial relationships that could be construed as a potential conflict of interest.

## Abbreviations

aHUS: Atypical HUS
HUS: Haemolytic Uraemic syndrome
FH: Factor H
FHR: Factor H-Related
FI: Factor I
RCA-I: *Ricinus Communis Agglutinin I*
SNA: *Sambucus Nigra Agglutinin*
SP: *Streptococcus pneumoniae*.
